# Prevalence and Risk Factors of Dengue Infection in Khanh Hoa Province, Viet Nam: A Stratified Cluster Sampling Survey

**DOI:** 10.2188/jea.JE20170090

**Published:** 2018-12-05

**Authors:** Vien Quang Mai, Trịnh Thị Xuan Mai, Ngo Le Minh Tam, Le Trung Nghia, Kenichi Komada, Hitoshi Murakami

**Affiliations:** 1 Nha Trang Pasteur Institute, Khanh Hoa, Viet Nam; 2 Department of Virology, Nha Trang Pasteur Institute, Khanh Hoa, Viet Nam; 3 Department of Laboratory Biosafety and Quality Assessment, Nha Trang Pasteur Institute, Khanh Hoa, Viet Nam; 4 Department of Vector Control and Border Quarantine, Nha Trang Pasteur Institute, Khanh Hoa, Viet Nam; 5 Bureau of International Health Cooperation, National Center for Global Health and Medicine, Tokyo, Japan

**Keywords:** dengue, Viet Nam, stratified cluster sampling survey

## Abstract

**Background:**

Dengue is a clinically important arthropod-borne viral disease with increasing global incidence. Here we aimed to estimate the prevalence of dengue infections in Khanh Hoa Province, central Viet Nam, and to identify risk factors for infection.

**Methods:**

We performed a stratified cluster sampling survey including residents of 3–60 years of age in Nha Trang City, Ninh Hoa District and Dien Khanh District, Khanh Hoa Province, in October 2011. Immunoglobulin G (IgG) and immunoglobulin M (IgM) against dengue were analyzed using a rapid test kit. Participants completed a questionnaire exploring clinical dengue incidence, socio-economic status, and individual behavior. A household checklist was used to examine environment, mosquito larvae presence, and exposure to public health interventions.

**Results:**

IgG positivity was 20.5% (urban, 16.3%; rural, 23.0%), IgM positivity was 6.7% (urban, 6.4%; rural, 6.9%), and incidence of clinically compatible dengue during the prior 3 months was 2.8 per 1,000 persons (urban, 1.7; rural, 3.4). For IgG positivity, the adjusted odds ratio (AOR) was 2.68 (95% confidence interval [CI], 1.24–5.81) for mosquito larvae presence in water pooled in old tires and was 3.09 (95% CI, 1.75–5.46) for proximity to a densely inhabited area. For IgM positivity, the AOR was 3.06 (95% CI, 1.50–6.23) for proximity to a densely inhabited area.

**Conclusions:**

Our results indicated rural penetration of dengue infections. Control measures should target densely inhabited areas, and may include clean-up of discarded tires and water-collecting waste.

## INTRODUCTION

Dengue fever (DF)/dengue hemorrhagic fever (DHF) is an arthropod-borne viral disease that is showing rapid global expansion. Over the past 5 decades, its global incidence has increased 30-fold, and it has shown geographic expansion to new countries and from urban to rural areas.^1^^,^^2^ A global modeling study estimates that 390 million dengue infections occurred in 2010, of which 96 million manifested with clinical or sub-clinical severity.^3^ Control program effectiveness is compromised largely due to the complexity and difficulty of establishing vector control. Moreover, vector resistance to insecticides has been reported at multiple sites.^4^ The effectiveness of insecticide-based strategies is not well established since few published studies have formally and rigorously assessed their impact on dengue incidence.^5^^,^^6^ Epidemiological studies are essential to properly focus prevention and control measures on high-risk groups, especially since the World Health Organization (WHO) issued its first position paper on the dengue vaccine in 2016. In this paper, the WHO recommends that countries only consider introducing the CYD-TDV dengue vaccine in geographic settings with a seroprevalence of ≥70% in the targeted age group. The vaccine is not recommended in areas where seroprevalence is <50%.^7^

According to the 2010 records of the WHO, Viet Nam reported 128,831 dengue cases, with 55 deaths. All four serotypes were circulating, with prominence of DEN-1 and DEN-2.^8^ DHF had substantial economic impact on families in southern Viet Nam.^9^ Two previous studies have investigated DF/DHF in Khanh Hoa Province, Viet Nam. In 2006, a household environment survey in Nha Trang City assessed 1,438 wet containers in 196 premises, and reported that 20% were positive for *Aedes aegypti* larvae and 8% for *Aedes aegypti* pupae, indicating favorable conditions for DF/DHF circulation and persistance.^10^ Additionally, a cohort study in 2005 to 2008 performed spatial analysis that included Nha Trang City and Ninh Hoa District and revealed that dengue risk was higher in rural areas compared to urban areas due to the lack of a piped water supply and a human population density within the critical range of 3,000 to 7,000 people/km^2^.^11^ A study examined how seasonality and climate factors were associated with DF incidence in four provinces, based on the surveillance data from 1994 to 2013, and revealed a significantly increased DF incidence between July and November compared to the incidence in February in Khanh Hoa Province.^12^ Among the four provinces (Hanoi in the north, Khanh Hoa in the central, Ho Chi Minh City and An Giang in the south), Khanh Hoa had the average incidence rate of 23.473 per 100,000 population (95% confidence interval [CI], 23.468–23.479), which was higher than those of the other three provinces. Increased incidence rates were observed in the second half of each year (from May through December), which is the rainy season, in all four provinces. From these findings, the endemicity of Khanh Hoa was considered high, while the seasonality of incidence was similar with other regions due to the synchronicity of the rainy season. Additionally, DF/DHF surveillance data from 2007 to 2010 in Nha Trang City, Ninh Hoa District, and Dien Khanh District revealed apparent epidemics in both 2007 and 2010, with the peak incidence occurring between July and October. However, population-based sero-epidemiology and individual risk factors for DF infection are not well documented in central Viet Nam, including in Khanh Hoa Province.

In our present study, we aimed to estimate the prevalence of dengue infection among residents in Nha Trang City and its vicinity in Khanh Hoa Province, central Viet Nam, a year after a large dengue epidemic in this region. The epidemic started in May 2010 and subsided in December 2010, during which time 3,401 cases were reported in Nha Trang City, Ninh Hoa District, and Dien Khanh District. Here we targeted the end of the seasonal peak of DF incidence to reflect the infection rate during the high season of the inter-epidemic year. We further set out to identify risk factors of infections, including those associated with socio-economic status, living environment, individual behavior, and exposure to public health interventions. Based on the present results, we have made recommendations for better dengue control in central Viet Nam.

## METHODS

### Study subjects and overall design

**Figure 1.**
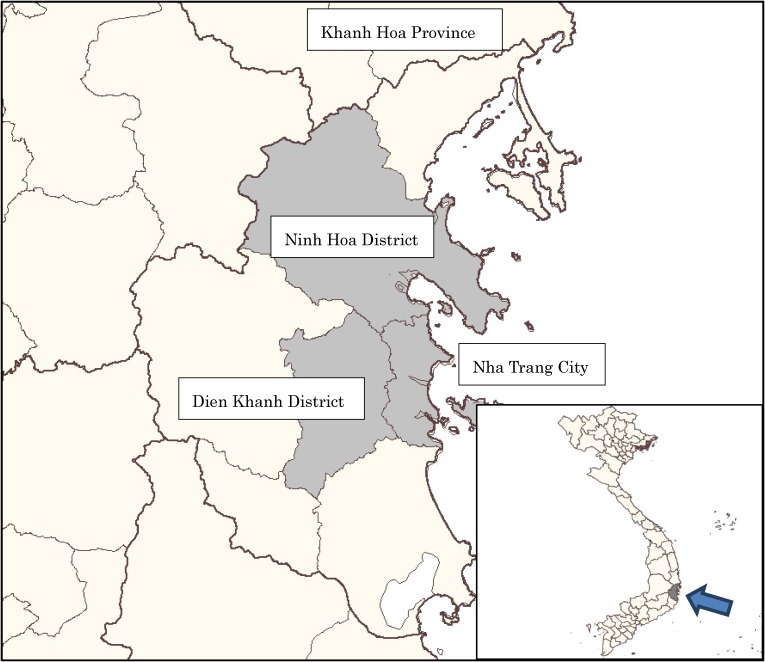
Map of Nha Trang City, Ninh Hoa District, and Dien Khanh District, Khahn Hoa Province, Viet Nam.

In October 2011, we conducted a population-based cross-sectional study. The study population comprised both males and females of 3–60 years of age, who were residents of the three selected districts: Nha Trang City (provincial capital), Ninh Hoa District, and Dien Khanh District (Figure 1). These districts form a continuous geographic region that includes both urban and rural areas according to the 2009 national census designation.^13^ Total population of the study site was 757,345 (Nha Trang City 367,484, Ninh Hoa District 241,173, and Dien Khanh District 148,688). The survey excluded children of <3 years of age and elderly residents of >60 years of age because local values did not condone serological testing of these age groups. Non-responding subjects were included in the survey dataset, but their sample weights were reallocated to other samples using a stratification variable (urban or rural area) for non-response adjustment of the sample weight.

We temporarily recruited staff of Nha Trang Pasteur Institute, Provincial and District Preventive Medicine Centers and Commune Health Centers to act as surveyors. Prior to survey implementation, they were provided orientation and instructions with regards to questionnaire administration, use of household observation list and dengue serological rapid testing. The study also included two supervision teams comprising staff of Nha Trang Pasteur Institute, Provincial Preventive Medicine Center and National Center for Global Health and Medicine (NCGM), Japan, which conducted visits to the survey teams while they were in the field.

### Sampling methods

We used the World Health Organization’s STEPS sample size calculator—assuming a confidence level of 95%, margin of error of 0.05, indicator baseline level of 25%, design effect of 2, expected response rate of 80%, and strata number of 2—and obtained a required sample size of 1,440. The 2009 national census estimated that the average number of household members in Khanh Hoa Province was 3.9, and that 84.4% of the population was between 3–60 years of age.^13^ Thus, we calculated that our study should include 437 households. To satisfy this requirement, we selected 15 households from 30 clusters to survey a total of 450 households.

We performed stratified two-step cluster sampling using enumeration areas (EAs) previously used for an in-depth survey of 15% of the nationwide households during the 2009 national census as primary sampling units. On average, each EA had 100 households. From the total of 110 EAs within Nha Trang City and the Ninh Hoa and Dien Khanh Districts, we selected 30 EAs by probability proportionate to size sampling. These 30 clusters were stratified by urban/rural division proportionate to the population size of each stratum. Urban areas included the provincial and district capital city/towns, as well as sub-district centers. We followed the 2009 national census designations of urban and rural EAs.^13^ Overall, 11 urban and 19 rural EAs were selected.

Within each selected EA, 15 households were randomly chosen from a household list generated by the General Statistics Office, Viet Nam. All members of the selected households who agreed to participate and who were between 3–60 years of age were included in the serological survey and the questionnaire, and their households were examined using the household observation list. They included persons with chronic conditions, such as hypertension, hepatitis, and asthma. Subjects of less than 15 years of age responded to the questionnaire in the presence of their primary care-givers.

### Serological testing

Both IgG and IgM against dengue were qualitatively tested using the Panbio Dengue Duo Cassette rapid test kit (Alere, Brisbane, Australia). This assay’s sensitivity is set such that individuals with primary dengue infection are positive for IgM and negative for IgG. In contrast, individuals with secondary or past dengue infections are positive for IgG, with or without a positive IgM result. The rapid test results were acquired in each household and were recorded in the questionnaire. The IgM results of the kit were pre-validated by the Nha Trang Pasteur Institute using 120 sera samples (60 dengue IgM positive and 60 dengue IgM negative) collected from dengue fever patients in 2010 and 2011. First, these 120 samples were retested using the IgM antigen-capture enzyme-linked immunosorbent assay (MAC-ELISA) following the protocol of the Armed Forces Research Institute of Medical Sciences (AFRIMS) to confirm those findings. Then the 120 samples were tested using the Panbio Dengue Duo Cassette following the manufacturer’s instructions. The Panbio Dengue Duo Cassette showed 95.0% sensitivity and 96.7% specificity in comparison with the MAC-ELISA results as the gold standard. The IgG results were not pre-validated because our main outcome indicator was IgM rather than IgG positivity. The positivity threshold of the kit for IgG is approximately ≥1:2,560 in hemagglutination-inhibition (HAI) assay and that for IgM is estimated to be around ≥40 units in MAC-ELISA following the AFRIMS protocol.^14^^,^^15^

### Questionnaire and observation list

We developed an individual questionnaire and a household observation list. The questionnaire included questions regarding the serological rapid test results, DF/DHF clinical manifestations (signs and symptoms) during the past 3 months, previous diagnosis as dengue fever in the lifetime, socio-economic status (eg, age, sex, education level, occupation, and permanent residents or migrants), individual behavior (eg, use of bed net, mosquito coil, repellent, insecticides, and mosquito racket; mosquito bite frequency and situations; and clothing habits), and exposure to dengue health education. The dengue health education was conducted by the local public health officers targeting the general public. The household observation list included items related to the household environment (eg, proximity to still water, densely inhabited areas, rice fields, and high and low vegetation bush), presence of mosquito larvae (eg, in water jars, old tires, fish ponds, flower vases, metal cans, sold rubbish collecting water, and water drainage ditches), and exposure to public health interventions (insecticide spraying and mosquito larvae control). The survey team members visually confirmed the household environment and the presence of mosquito larvae and filled the observation list. Both the questionnaire and the observation list were pre-tested and amended to ensure that laypersons clearly understood the queries in the questionnaire and that the observation list was easy to use.

### Statistical analysis

Data analyses fully accounted for effects of sampling design both by weighing the samples and by adjusting the standard error (SE) to reflect intraclass correlation. Sample weights were calculated as the inverse of sample selection probabilities. To calculate sample selection probabilities, we multiplied the EA selection probabilities (EA total population/sampling interval that is the total population of the city and two districts divided by 30) and household selection probabilities within the selected EAs (15/total number of households in a given EA). After calculating base weights for samples, we performed non-response adjustment using urban or rural area as a frame. Then, the weights were post-stratified according to the ratio of urban and rural populations in the surveyed city and two districts. We did not set households as the secondary sampling unit because the study included all eligible household members, such that the selection probability was 1 for all of them. Therefore, our statistical analysis fully accounted for the intra-class correlation within selected villages but did not account for that within selected households.

We performed point and range estimations of IgG and IgM positivity and of the incidence of clinically compatible DF/DHF, accounting for the sampling design. Clinically compatible DF/DHF was defined as the experience of acute febrile illness in the past 3 months, along with either IgG or IgM positivity, and two or more of the following manifestations: headache, retro-orbital pain, myalgia, arthralgia, rash, and hemorrhagic manifestations. We explored associations between IgG and IgM positivity as well as clinical DF/DHF and socio-economic, individual behavioral, and household environmental factors. We also analyzed the impact of exposure to public health interventions. We initially performed univariate logistic regression analyses with IgG, IgM, and clinical DF/DHF set as dependent variables, and with socio-economic, individual behavioral, household environmental, and public health intervention factors set as independent variables. We then constructed multiple logistic regression models, including the independent variables that showed significant association with the dependent variables. For each analysis, we computed the post-survey design effect (deff). Statistical significance was set at *P* < 0.05. Data analyses were performed using STATA12 (StataCorp LP, College Station, TX, USA).

### Ethical considerations

The survey protocol was ethically reviewed and approved both by the Ethics Committee of NCGM and by the Science Committee of Khanh Hoa Province. Subjects were informed of their freedom to decline and other rights in a non-coercive environment and were asked to sign a consent form prior to participation.

## RESULTS

### Characteristics of study subjects

**Figure 2.**
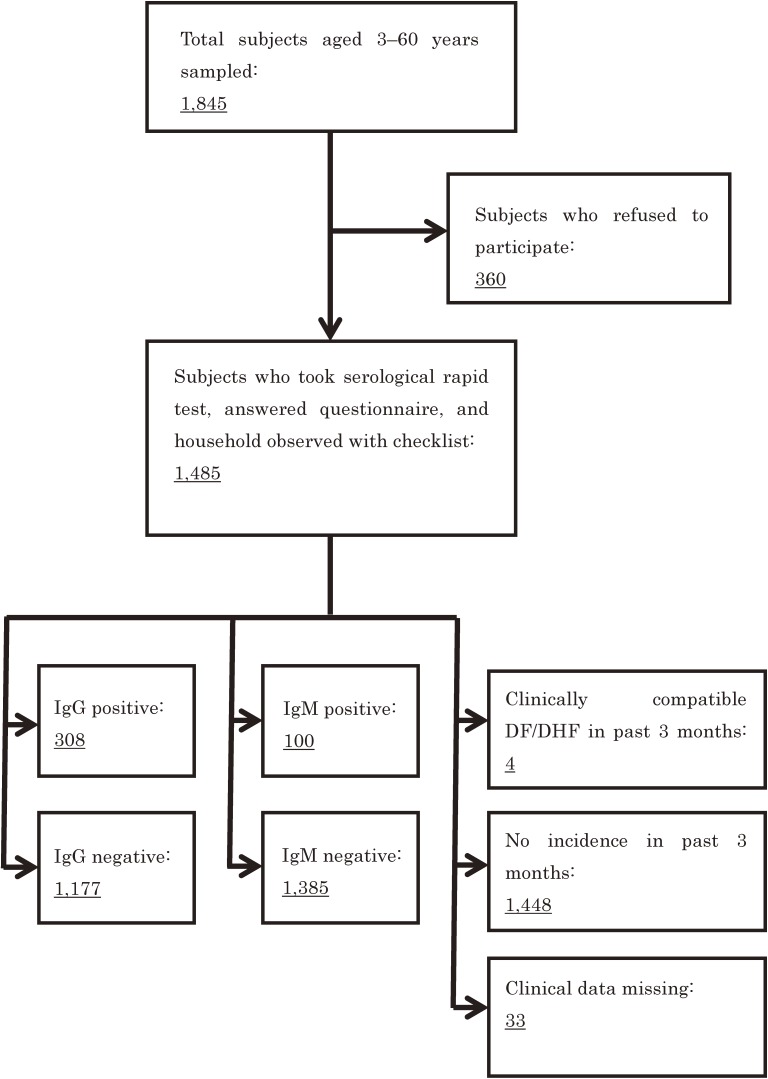
Flow chart of subjects of the survey in Nha Trang City, Ninh Hoa District, and Dien Khanh District, Khahn Hoa Province, Viet Nam. IgG, immunoglobulin G; IgM, immunoglobulin M; DF, dengue fever; DHF, dengue hemorrhagic fever.

A total of 1,845 persons were invited to participate, of whom 1,483 (80.4%) agreed to undergo the serological rapid test and answer the questionnaire. Altogether, 450 households were sampled and visited. In seven households, all household members refused to participate; thus, 443 households participated in the study. Figure 2 shows a flow chart of the study subjects. Table 1 presents the key characteristics of the subjects. The mean age was 29.14 years, and there were similar proportions of males and females. With regards to educational level, the largest proportion of participants were secondary school graduates. Common job categories included students, farmers, housewives, industry workers, and shop/restaurant workers.

**Table 1.** Characteristics of surveyed residents of Nha Trang City, Ninh Hoa District, and Dien Khanh District, Khanh Hoa Province, Viet Nam

Total number of subjects who took serological test	1,485
Socioeconomic status	
Age, years, mean (SD)	29.14 (15.63)
Female sex, *n* (%)	765 (52.1)
Urban or rural area (residence)	
Urban, *n* (%)	543 (36.6)
Rural, *n* (%)	942 (63.4)
Educational level, *n* (%)	
Preschool	58 (4.0)
Primary school	374 (25.6)
Secondary school	582 (39.9)
High school	317 (21.7)
University/college	60 (4.1)
Others (mostly non-educated)	69 (4.7)
Job, *n* (%)	
Preschool	58 (3.9)
Students	407 (27.4)
Housewife (no work)	181 (12.2)
Farmer	244 (16.5)
Fisherman	48 (3.2)
Industry worker	162 (10.9)
Craft man	56 (3.8)
Office worker	52 (3.5)
Shop/restaurant worker	117 (7.9)
Retired	10 (0.7)
Others	148 (10.0)
Household environment, *n* (%)	
Proximity to densely inhibited area (<50 m)	922 (62.1)
Proximity to rice field (<50 m)	398 (26.8)
Proximity to high vegetation bush (<50 m)	890 (59.9)
Proximity to low vegetation bush (<50 m)	910 (61.3)

SD, standard deviation.

### Prevalence of IgG and IgM positivity and incidence of clinically compatible DF/DHF

Table 2 presents the prevalence of dengue IgG/IgM positivity and the incidence of clinically compatible DF/DHF among survey subjects. The estimated total IgG positivity was 20.5% (95% CI, 13.6–27.4%), with an estimated urban prevalence of 16.3% (95% CI, 7.2–25.3%) and estimated rural prevalence of 23.0% (95% CI, 12.9–33.2%). The estimated total IgM positivity was 6.7% (95% CI, 4.0–9.4%), with an estimated urban prevalence of 6.4% (95% CI, 2.2–10.5%) and estimated rural prevalence of 6.9% (95% CI, 3.2–10.7%). The estimated total incidence of clinically compatible DF/DHF during the past 3 months was 2.8 cases per 1,000 persons (95% CI, 0.1 to 5.5), with an estimated urban incidence of 1.7 cases per 1,000 persons (95% CI, −2.1 to 5.6) and estimated rural incidence of 3.4 cases per 1,000 persons (95% CI, 0.5 to 7.3). Overall, the rates were higher in rural compared to urban areas, although these differences were not statistically significant. These rates did not significantly differ between different age strata or between sexes. Table 3 shows the prevalence of IgG/IgM positivity by age strata. Neither IgG nor IgM prevalence showed a trend of increasing or decreasing across age strata. However, the youngest age stratum (3–9 years) showed the lowest positivity for both IgG and IgM. Individuals who had ever been diagnosed as dengue showed significantly higher IgG positivity (33.3%) than those who had not (19.1%, *P* = 0.003).

**Table 2.** Prevalence of dengue IgG/IgM positivity and incidence of clinically compatible dengue fever/dengue hemorrhagic fever in past three months among survey subjects in Khanh Hoa Province, Viet Nam

Stratification (urban or rural)	*N*	Number of positive cases	Prevalence (%)	95% CI (%)	Standard error	Design effect
**Dengue IgG positive**						
Urban	543	90	16.3	7.2–25.3	0.041	6.545
Rural	942	218	23.0	12.9–33.2	0.048	12.324
**Total**	**1,485**	**308**	**20.5**	**13.6**–**27.4**	**0.034**	**10.361**
**Dengue IgM positive**						
Urban	543	35	6.4	2.2–10.5	0.018	3.106
Rural	942	65	6.9	3.2–10.7	0.018	4.683
**Total**	**1,485**	**100**	**6.7**	**4.0**–**9.4**	**0.013**	**4.112**

Stratification (Urban or rural)	*N*	Number of compatible cases	Incidence (/1,000 persons-3 months)	95% CI	Standard error	Design effect

**Clinically compatible dengue fever or dengue hemorrhagic fever with either IgG or IgM positivity in past 3 months**						
Urban	531	1	1.7	−2.1–5.6	0.002	0.898
Rural	921	3	3.4	0.5–7.3	0.002	0.905
**Total**	**1,452**	**4**	**2.8**	**0.1**–**5.5**	**0.013**	**0.901**

CI, confidence interval; IgG, immunoglobulin G; IgM, immunoglobulin M.

**Table 3.** Prevalence of dengue IgG/IgM positivity by age strata among survey subjects in Khanh Hoa Province, Viet Nam

Age strata	*N*	IgG positivity^a^			IgM positivity^a^		
	
		No. of positive cases	Prevalence (%)	95% CI (%)	No. of positive cases	Prevalence (%)	95% CI (%)
3–9 years	175	25	13.8	7.1–20.5	7	4.1	1.3–6.9
10–19 years	318	76	23.7	12.8–34.5	17	5.3	2.1–8.6
20–29 years	275	67	24.3	15.6–33.1	23	8.2	3.5–12.9
30–39 years	243	47	19.2	11.5–26.8	15	6.3	2.2–10.4
40–49 years	307	68	21.8	14.5–29.0	28	9.1	5.6–12.6
50–60 years	161	24	14.8	7.0–22.7	10	6.2	2.5–9.9
Total	1,479^b^	307^c^	20.5	13.6–27.4	100	6.7	4.0–9.4

CI, confidence interval; IgG, immunoglobulin G; IgM, immunoglobulin M.

^a^Percentage figures are the estimated prevalence of all population aged 3–60 years in Nha Trang City, Ninh Hoa District and Dien Khanh District taking into account sample weights and intra-class correlation, therefore not equal with the simple division of the numbers of cases by *N*.

^b^Six cases are missing age information.

^c^One case is missing age information.

### Risk factors associated with IgG and IgM positivity and clinically compatible DF/DHF

Table 4 presents the results of univariate and multiple logistic regression analyses of the associations between dengue IgG positivity and various factors. Univariate analysis revealed that IgG positivity was associated with job, types of shirts worn in the evening and during sleep, frequency of dengue education received in past 2 years, mosquito larvae presence in water pooled in old tires in household compound, and household proximity to a densely inhabited area (<50 m). These factors were included in a multiple logistic regression model. In this model, IgG positivity remained significantly associated with dengue education, mosquito larvae presence in water pooled in old tires, and proximity to a densely inhabited area. The adjusted odds ratio (AOR) was 2.21 (95% CI, 1.27–3.84) for receiving dengue education 3–4 times in the past 2 years compared to 5 times. The AOR was 2.68 (95% CI, 1.24–5.81) for mosquito larvae presence in water pooled in old tires and was 3.09 (95% CI, 1.75–5.46) for proximity to a densely inhabited area.

**Table 4.** Univariate and multiple logistic regression analyses of associations between dengue IgG positivity and various factors among studied subjects in Nha Trang City, Ninh Hoa District, and Dieh Khanh District, Khan Hoa Province, Viet Nam

Independent variables	*N*	Univariate analysis				Multiple logistic regression analysis			
	
		OR (95% CI)	*P*	SE	Deff	AOR (95% CI)	*P*	SE	Deff
Job									
Student	407	Reference	—	—	—	Reference	—	—	—
Preschool	58	0.59 (0.29–1.17)	0.123	0.20	0.69	0.44 (0.16–1.22)	0.109	0.22	0.94
Housewife (no work)	181	0.93 (0.60–1.44)	0.752	0.20	0.91	0.82 (0.49–1.38)	0.446	0.21	0.93
Farmer	244	0.48 (0.25–0.89)	0.021^*^	0.14	1.64	0.67 (0.37–1.22)	0.187	0.20	1.20
Fisherman	48	1.33 (0.59–2.99)	0.471	0.53	1.23	0.87 (0.33–2.30)	0.767	0.41	1.04
Industry worker	162	1.41 (0.89–2.23)	0.134	0.32	1.09	0.98 (0.61–1.56)	0.915	0.22	0.78
Craft man	56	0.20 (0.04–0.99)	0.048^*^	0.16	1.56	0.19 (0.03–1.03)	0.054	0.16	1.74
Office worker	52	1.18 (0.63–2.22)	0.593	0.36	0.80	0.96 (0.46–1.97)	0.902	0.34	0.80
Shop/restaurant worker	117	1.03 (0.53–2.01)	0.932	0.34	1.63	0.78 (0.36–1.68)	0.506	0.29	1.65
Retired	10	0.36 (0.04–3.17)	0.345	0.38	1.04	0.37 (0.05–3.04)	0.343	0.38	0.96
Others	148	1.82 (1.01–3.28)	0.046^*^	0.52	1.82	1.37 (0.73–2.59)	0.317	0.43	1.56
Types of shirts worn in the evening (5–10 pm)									
Short-sleeve	1100	Reference	—	—	—	Reference	—	—	—
Long-sleeve	103	0.57 (0.28–1.18)	0.124	0.20	1.38	0.62 (0.25–1.56)	0.301	0.28	1.28
Tank top	211	0.87 (0.57–1.35)	0.529	0.18	1.20	1.00 (0.59–1.72)	0.987	0.26	1.03
Naked	67	1.91 (1.21–3.02)	0.007^*^	0.43	0.68	1.39 (0.62–3.10)	0.413	0.54	0.88
Types of shirts worn during sleep									
Short-sleeve	917	Reference	—	—	—	Reference	—	—	—
Long-sleeve	39	0.34 (0.09–1.30)	0.111	0.22	1.16	0.90 (0.22–3.61)	0.877	0.61	0.83
Tank top	376	0.97 (0.62–1.51)	0.881	0.21	1.96	1.00 (0.57–1.73)	0.987	0.27	1.79
Naked	144	1.83 (1.17–2.86)	0.010^*^	0.40	1.20	1.32 (0.68–2.57)	0.403	0.43	1.24
Dengue education in past 2 years									
More than 5 times	403	Reference	—	—	—	Reference	—	—	—
3–4 times	252	2.27 (1.23–4.21)	0.011^*^	0.68	2.64	2.21 (1.27–3.84)	0.007^*^	0.59	1.90
1–2 times	373	0.72 (0.39–1.33)	0.280	0.22	2.36	0.96 (0.55–1.66)	0.869	0.26	1.67
None	183	1.55 (0.59–4.12)	0.362	0.74	5.06	1.82 (0.75–4.42)	0.176	0.79	3.60
Mosquito larvae observed in water pooled in old tires									
No	1457	Reference				Reference	—	—	—
Yes	28	2.49 (1.31–4.74)	0.007^*^	0.78	0.59	2.68 (1.24–5.81)	0.014^*^	1.01	0.61
Densely inhabited area within 50 m of the household									
No	563	Reference	—	—	—	Reference	—	—	—
Yes	922	3.89 (2.15–7.04)	0.000^*^	1.13	3.02	3.09 (1.75–5.46)	0.000^*^	0.86	1.96

AOR, adjusted odds ratio; CI, confidence interval; Deff, design effect; *N*, number of cases; OR, odds ratio; SE, standard error.

^*^P < 0.05.

Table 5 presents the results of univariate and multiple logistic regression analyses of associations between dengue IgM positivity and various factors. Univariate analysis revealed that IgM positivity was associated with educational status, job, types of shirts worn in the evening and during sleep, and proximity to a densely inhabited area (<50 m). In the generated multiple logistic regression model, IgM positivity remained significantly associated with only proximity to a densely inhabited area. The AOR was 3.06 (95% CI, 1.50–6.23) for proximity to a densely inhabited area. Dengue seropositivity was not associated with any individual behavior variables, including use of bed net, mosquito coil, repellent, insecticides, and mosquito racket.

**Table 5.** Univariate and multiple logistic regression analyses of associations between dengue IgM positivity and various factors among studied subjects in Nha Trang City, Ninh Hoa District, and Dieh Khanh District, Khan Hoa Province, Viet Nam

Independent variables	*N*	Univariate analysis				Multiple logistic regression analysis			
	
		OR (95% CI)	*P*	SE	Deff	AOR (95% CI)	*P*	SE	Deff
Educational status									
Secondary school	582	Reference	—	—	—	Reference	—	—	—
Primary school	374	0.96 (0.61–1.50)	0.843	0.21	0.54	1.12 (0.68–1.84)	0.654	0.27	0.57
High school	317	1.85 (1.13–3.01)	0.016^*^	0.44	0.82	1.56 (0.91–2.67)	0.104	0.41	0.90
University/college	60	1.26 (0.47–3.33)	0.635	0.60	0.79	1.08 (0.32–3.61)	0.898	0.64	1.05
Preschool	58	0.64 (0.13–3.04)	0.563	0.49	1.05	1 (omitted)^a^	—	—	—
Others	69	2.44 (1.01–5.94)	0.048^*^	1.06	1.28	2.37 (0.97–5.76)	0.057	1.03	1.16
Job									
Student	407	Reference	—	—	—	Reference	—	—	—
Preschool	58	0.59 (0.12–3.00)	0.513	0.47	1.12	0.97 (0.17–5.53)	0.973	0.82	1.16
Housewife (no work)	181	0.96 (0.37–2.48)	0.926	0.44	1.44	0.82 (0.29–2.33)	0.696	0.42	1.30
Farmer	244	0.41 (0.15–1.11)	0.077	0.20	1.09	0.70 (0.27–1.82)	0.448	0.33	0.97
Fisherman	48	1.99 (0.58–6.77)	0.261	1.19	1.34	1.57 (0.40–6.13)	0.502	1.04	1.33
Industry worker	162	2.08 (1.03–4.21)	0.043^*^	0.72	1.11	1.78 (0.84–3.74)	0.126	0.65	1.12
Craft man	56	0.59 (0.14–2.61)	0.476	0.43	0.91	0.61 (0.13–2.80)	0.515	0.45	0.92
Office worker	52	2.03 (0.78–5.29)	0.141	0.95	0.92	1.23 (0.43–3.52)	0.690	0.63	0.92
Shop/restaurant worker	117	0.97 (0.47–1.99)	0.932	0.34	0.60	0.85 (0.39–1.86)	0.670	0.33	0.67
Retired	10	1.56 (0.19–12.62)	0.664	1.59	0.93	1.69 (0.17–16.39)	0.640	1.87	0.95
Others	148	2.21 (1.24–3.93)	0.009^*^	0.62	0.73	1.57 (0.85–2.92)	0.145	0.47	0.73
Types of shirts worn in the evening (5–10 pm)									
Short-sleeve	1100	Reference	—	—	—	Reference	—	—	—
Long-sleeve	103	0.49 (0.13–1.83)	0.275	0.31	1.32	0.53 (0.15–1.88)	0.314	0.33	1.33
Tank top	211	0.83 (0.44–1.58)	0.562	0.26	0.92	0.62 (0.35–1.12)	0.109	0.18	0.52
Naked	67	2.53 (1.38–4.65)	0.004^*^	0.75	0.67	1.23 (0.55–2.72)	0.605	0.48	0.76
Types of shirts worn during sleep									
Short-sleeve	917	Reference	—	—	—	Reference	—	—	—
Long-sleeve	39	0.53 (0.07–3.88)	0.515	0.51	0.97	1.06 (0.14–8.26)	0.957	1.06	1.07
Tank top	376	1.50 (0.93–2.44)	0.094	0.35	0.93	1.60 (0.94–2.72)	0.084	0.42	0.86
Naked	144	2.88 (1.52–5.46)	0.002^*^	0.90	1.20	1.96 (0.77–4.95)	0.150	0.89	1.61
Densely inhabited area within 50 m of the household									
No	563	Reference	—	—	—	Reference	—	—	—
Yes	922	3.60 (1.77–7.31)	0.001^*^	1.25	1.47	3.06 (1.50–6.23)	0.003^*^	1.06	1.10

AOR, adjusted odds ratio; CI, confidence interval; Deff, design effect; *N*, number of cases; OR, odds ratio; SE, standard error.

^a^Omitted because of collinearity.

^*^P < 0.05.

Clinically compatible DF/DHF was only significantly associated with mosquito larvae presence in water pooled in old tires. The crude odds ratio was 17.42 (95% CI, 1.63–186.34; *P* = 0.020; SE = 20.15; Deff = 0.92).

Mosquito larvae presence was confirmed in flower vases in 201 cases (13.6%), solid rubbish collecting rain water in 187 cases (12.6%), water drainage ditches in 131 cases (8.8%), uncovered water jars in 97 cases (6.5%), old tires in 28 cases (1.9%), metal cans in 14 cases (0.9%), and ponds in 10 cases (0.7%). Among these larvae presence sites, only old tires showed statistically significant association with IgG positivity and clinically compatible DF/DHF.

## DISCUSSION

At the time of this study, the dengue seroprevalence in Nha Trang City, Ninh Hoa District, and Dien Khanh District was equally high in rural and urban areas, indicating widespread rural penetration of this disease. Proximity of a household to a densely inhabited area was a common risk factor for both IgG and IgM positivity.

Our present study has a number of limitations. First, serological confirmation was performed using a qualitative rather than quantitative method. Although pre-validation testing demonstrated high sensitivity and specificity of IgM results, the results may include some degree of false-positive and false-negative results. Second, 362 individuals were sampled but refused to participate. These non-respondents may have contributed to over-representation of certain characteristics among the survey participants. Third, it is difficult to determine whether IgG positivity reflects only past dengue infection or if it reflects a recent secondary infection associated with the current risk factors identified in this study. Participants with a dengue diagnosis at any point in their lifetime showed significantly higher IgG positivity than those never diagnosed as dengue, indicating that the IgG positivity likely reflected past infections and may not be a good indicator of dengue exposure during the survey period. Fourth, despite the targeting on the end of the seasonal peak of DF incidence, the survey could identify only four clinically compatible DF cases. The limited number of clinically compatible cases hindered the analysis of risk factors for clinical DF/DHF incidence.

Historically, DF/DHF has been considered a disease of urban populations; however, ample reports demonstrate dengue transmission in rural settings.^3^^,^^16^^–^^18^ We hypothesized that the IgG and IgM positivity and the incidence of clinically compatible DF/DHF in rural area were comparable to those in urban area due to rural penetration of dengue. Major reasons for the spread of dengue to rural areas include increased transport contact, mobility, and the spread of peri-urbanization.^3^^,^^19^^,^^20^ Our study area included both urban and rural areas, with the latter being generally peri-urban in nature and, thus, prone to continued dengue transmission.

The dengue IgG positivity identified in our study was relatively lower than the results in neighboring areas in Southeast Asia. We presumed the IgG positivity of 25% based on a preceding study in Long Xuen, An Giang Province, Viet Nam.^21^ A dengue IgG serosurvey in Malaysia in 2008 reported dengue seropositivity in 91.6% of 1,000 adults of 35–74 years of age.^18^ In a serosurvey of 961 primary schoolchildren in Binh Thuan Province, Viet Nam, the IgG positivity rate was 65.7%.^22^ Three seroprevalence surveys have been conducted in Singapore, reporting IgG seroprevalence rates of 50.5% among 3,995 healthy adult blood donors in 2009–2010,^23^ 59% among 4,152 participants in a 2004 national health survey,^24^ and 45% among 298 staff and visitors of a large public hospital in 2002.^25^ All of these previously reported prevalence rates far exceed the rates demonstrated in our present study. The presently determined relatively low IgG prevalence rate may be explained by the period of low precipitation from January to August in Khanh Hoa Province. Similar to our present findings, IgG seroprevalence rates of 19.6–27.2% were reported from a dynamic school-based cohort of 2–15 year olds in Long Xuen, An Giang Province in southern Viet Nam from 2003 to 2007.^21^

In contrast to the IgG results, the dengue IgM positivity determined in our study was relatively higher than the results in neighboring areas in Southeast Asia. A 2001 community-based survey in northern Thailand reported an estimated IgM seroconversion rate of 6.5%,^26^ which is comparable with our result. The blood donor serosurvey in Singapore in 2009–2010 revealed an IgM seropositivity rate of 2.8%,^23^ while the Singapore national health survey in 2004 reported a rate of 2.6%.^24^

The incidence of clinically compatible dengue found in our study was relatively lower than the results in neighboring areas. We estimated that the incidence of clinically compatible dengue with either IgG or IgM positivity was 2.8 per 1,000 persons for the months of July, August, and September, which was the peak dengue season during prior epidemic years. Annualized, this represents 11.2 cases per 1,000 persons. A school-based cohort study in Long Xuyen, Viet Nam, reported an acute dengue incidence ranging from 16.9 per 1,000 persons in 2005 to 40.4 per 1,000 persons in 2007.^21^ Based on active surveillance data from Cambodia during 2006–2008, the estimated annual incidence of clinical DF/DHF with IgM positivity was 13.7 per 1,000 population during an inter-epidemic year and 57.8 per 1,000 population during an epidemic year.^27^

Our present results showed that dengue infection rates did not differ between sexes, which is common across other community-based serological surveys.^18^^,^^22^^,^^24^^,^^28^ However, male predominance of dengue infection has been reported in northern Thailand^26^ and Singapore.^23^ Although we hypothesized higher IgG positivity among older age groups, our present results showed no statistically significant seroprevalence differences across different age strata. A study of dengue outbreak in El Salvador also found no age-related differences.^29^ However, other studies have shown varying infection rates across different age strata. Higher infection rates have been described among older adults in a number of studies in Viet Nam,^22^ in other countries in Southeast Asia,^23^^–^^26^ and in Latin America.^28^^,^^30^

We identified household proximity to a densely inhabited area (within 50 m) as a significant risk factor for both IgG and IgM positivity. Similarly, household proximity to a densely inhabited village center (within 200 m) was identified as a risk factor in northern Thailand.^26^ Entomological studies report that the flight range of *Aedes aegypti* and *Aedes albopictus* is limited to within 100 m,^31^^–^^33^ which partly explains why proximity to a densely populated region is associated with higher risk of dengue infection. Notably, even remote areas contain densely inhabited areas that posed increased risk of dengue infection. As shown in Table 1, 62.1% of the total survey subjects and 47.3% of the subjects in rural areas resided within close proximity to the densely inhabited areas. These results support the targeting of control measures to densely inhabited areas, particularly in rural areas.

Our results showed that IgG positivity was more common among subjects who had received dengue health education 3–4 times during the past 2 years compared to those who had received such education over 5 times. However, the positivity rates did not significantly differ between the groups who answered >5 times compared to 1–2 times or none. This odd result is likely derived from recall bias. Those who answered 1–2 times or none may represent subjects who received dengue education long ago and cannot recall such education, regardless of their dengue infection status. Furthermore, the difference between the groups who answered >5 times and none might have failed to reach statistical significance due to the smaller number of subjects who answered none compared to other frequencies.

Our data also revealed that mosquito larvae presence in water pooled in old tires within household compounds was associated with IgG positivity and clinical DF/DHF. However, the association between larvae presence in old tires and clinical DF/DHF was based only on four clinically compatible cases; therefore, this finding may be very prone to coincidence. In a study of primary school children in Binh Thuan Province, Viet Nam, dengue IgG positivity was significantly associated with pit latrine usage, the presence of discarded cans in a domestic environment, and keeping pigs.^22^ One study presented the hypothesis that a dengue outbreak in El Salvador in 2000 was associated with the presence of mosquito-infested solid waste and determined that infection was indeed associated with the presence of mosquito larvae in discarded cans, plastic containers, and tire casings in the home environment.^29^

Over past decades, emphasis has been placed on the use of ultra-low-volume insecticide space sprays for adult mosquito control, which is now considered a relatively ineffective approach for controlling *Aedes aegypti*.^20^^,^^34^ Pilot studies in Viet Nam have proven the hypothesis that the treatment of water storage vessels with copepods to kill larvae,^35^^,^^36^ and of covering vessels with Olyset^®^ Net lids with pyriproxyfen^37^ are effective. The water in our study area had not been treated with larvicide due to local concerns regarding toxicity. Physical reduction of larval development sites by disposing of discarded or unnecessary water containers is easier to implement than other mosquito control measures.

### Conclusions

Our present results indicate the widespread rural penetration of dengue infections within the studied area. Control measures should target densely inhabited areas and may include clean-up of discarded tires and water-collecting solid waste.
